# Phosphine poisoning in free‐range local chickens: a case report

**DOI:** 10.1002/vms3.100

**Published:** 2018-03-14

**Authors:** Issa A. Muraina, Olusola O. Oladipo, Olatunde B. Akanbi, Johnson J. Shallmizhili, Moses D. Gyang, Gabriel O. Ijale, Felix P. Govwang, Aliyu A. Atiku

**Affiliations:** ^1^ Toxicology Section, Biochemistry Division National Veterinary Research Institute Vom Nigeria; ^2^ Central Diagnostic Laboratory National Veterinary Research Institute Vom Nigeria; ^3^ Department of Veterinary Pathology Faculty of Veterinary Medicine University of Ilorin Ilorin Nigeria; ^4^ Unity Veterinary Clinic Jos Nigeria

**Keywords:** Acute toxicity, chickens, phosphide, phosphine, poisoning, rodenticide

## Abstract

Phosphine poisoning is rarely reported in poultry, and its diagnosis is a great challenge for veterinary toxicologists and pathologists. A case of phosphine toxicosis in local, free range chickens is reported. Fourteen dead chickens (age ≥6 months old) were presented to the veterinary clinic for necropsy. The history revealed that the chickens were normally fed with guinea corn grains, but were suspected to have been fed boiled rice laced with poison. The clinical signs observed were ruffled feathers, somnolence, anorexia and high mortality. The disease ran a 2‐day course with mortality pattern of four chickens the first day, six overnight and 14 the following day. Necropsy findings showed generalized vascular congestion and haemorrhage in the lungs and visceral organs, with the crop and gizzard filled with guinea corn and rice grains and greenish‐yellow faecal material in the intestinal lumen. The presence of widespread congestion and petechial haemorrhages on visceral organs with the microscopic pulmonary congestion, and diffuse intraparabronchial presence of air sac macrophages, strongly suggested an acute toxic cause of death. Chemical tests on the crop contents of the dead chickens were positive for phosphine gas. This report will contribute to a better understanding of the clinical signs and lesions presented in cases of acute phosphide rodenticide exposure in domestic chickens, with a brief review of the forensic literature.

## Introduction

Traditionally, there have been many constraints on family poultry production in many rural areas of Africa. Village or local poultry production is a low input system with little investment in disease control and prevention, feed supplementation and housing, resulting in low output in production (Klos *et al*. 2004). Such chickens scavenge for meal and sources of feed like human food and post‐harvest wastes and materials from the environment. This method of raising local chickens and feeding habits expose predispose them to obnoxious substances like rodenticides and other poisonous substances in their environment.

Rodenticides can be obtained easily from stores in Nigeria and may contain zinc phosphide (Zn_3_P_2_) or aluminium phosphide (AlP). Both phosphides liberate phosphine gas (PH_3_) on contact with either atmospheric moisture or hydrochloric acid in the stomach (Guale *et al*. 1994; Tiwary *et al*. 2005). Phosphine gas is highly toxic to both animals and humans. It is a colourless and odourless gas in its pure form, but produces a foul odour of garlic or decaying fish in the presence of substituted phosphines and diphosphines (Chugh 1992). Upon ingestion of Zn_3_P_2_ or AlP, PH_3_ is released and rapidly absorbed throughout the gastrointestinal tract, and oxidized to oxyacid, causing systemic toxic effects in the heart, lungs, kidneys and liver with manifestations of serious cardiac arrhythmias, intractable shock, metabolic acidosis and pulmonary oedema (Hakimoglu *et al*. 2015). Phosphine is excreted in the urine as hypophosphite, and through the lungs in the unchanged form (Gurjar *et al*. 2011) causing further toxicosis through inhalation in subjects exposed to the breath or vomitus of poisoned animals or humans (Easterwood *et al*. 2010; CDC 2012, O'Malley *et al*. 2013; Lodde *et al*. 2015).

The signs of phosphine poisoning are instantaneous but non‐specific, depending on the dose, route of entry and time lapse since exposure to poison. Following oral ingestion, signs of toxicity usually develop within a few minutes. In animals, signs may include loss of appetite, nausea, vomiting (which may be bloody), abdominal pain, diarrhoea, lethargy, incoordination, convulsions, paralysis, coma and death (Knight 2007). In humans, symptoms may include headaches, shortness of breath, nausea, vomiting and dizziness. More severe symptoms, including gastrointestinal, respiratory distress, convulsions and death with renal and hepatic failure and disseminated intravascular coagulation can occur with severe phosphine poisoning (Sciuto *et al*. 2016).

A clinical diagnosis is usually based on suspicion, history of poisoning, identification of poison material or package, clinical signs, necropsy and histopathological changes (Nagy *et al*. 2015). Confirmation is by chemical analysis for presence of phosphine using the simple silver nitrate‐impregnated paper test on gastric content or on breath and gas chromatographic analysis of gastric contents (Tripathi *et al*. 1992). The silver nitrate paper test is based on the property of PH_3_ to reduce silver nitrate into silver which gives a black colour on the filter paper.

There are no specific antidotes for PH_3_ poisoning. Recent therapeutic measures indicate that a combination of glucagon, digoxin, antioxidants (Oghabian & Mehrpour 2016; Oghabian *et al*. 2016), activated charcoal or boric acid (Tehrani *et al*. 2013; Soltani *et al*. 2016) have shown promise in affected human patients. Other supportive therapies include gastric lavage with diluted potassium permanganate, coconut oil, sodium bicarbonate and intravenous magnesium sulphate (Agrawal *et al*. 2015) and castor oil (Shakoori *et al*. 2016). In the present study, we report for the first time a case of phosphine gas poisoning in free range local chickens in Nigeria.

## Case report

### Case history

The case involved a flock of 70 village chickens of mixed ages and sexes, reared on free range, but provided with shelter during the night and extreme weather conditions. On February 15th, 2016, four chickens were presented to Unity Veterinary Clinic, Jos, Nigeria for necropsy. History revealed that the farmer had lost about 10 chickens the previous day, while mortality rose to 14 the following day. History further revealed that the chickens were fed on guinea corn and scavenges in the environment. The chickens were also fed on boiled rice suspected to be laced with poison by a neighbour for the purpose of controlling rats. The diagnostic plan included postmortem, histopathological and toxicological examination, and a farm visit to examine the remaining chickens, environment and interview the farmer.

Tissue samples including the crop, intestines, liver, kidneys, spleen, lungs and feed samples were submitted to the toxicology section of NVRI, Vom, while tissue sections of the intestines, liver, kidneys, spleen and lungs were submitted to the Central Diagnostic Laboratory for histopathological examinations. On visiting the farm house, it was discovered that the birds were free range, apparently healthy and provided with shelter, while some were seen roaming about and mingling with pigeons. Mortality had ceased, suggesting that the case was a hyper‐acute condition in the chickens that had been fed the boiled rice. An interview with the farmer during farm visit revealed recent history of rodenticide rice bait for rodent control in the environment. The rodenticide container revealed zinc phosphide as a major component on the label.

### Pathological examination

**Figure 1:**
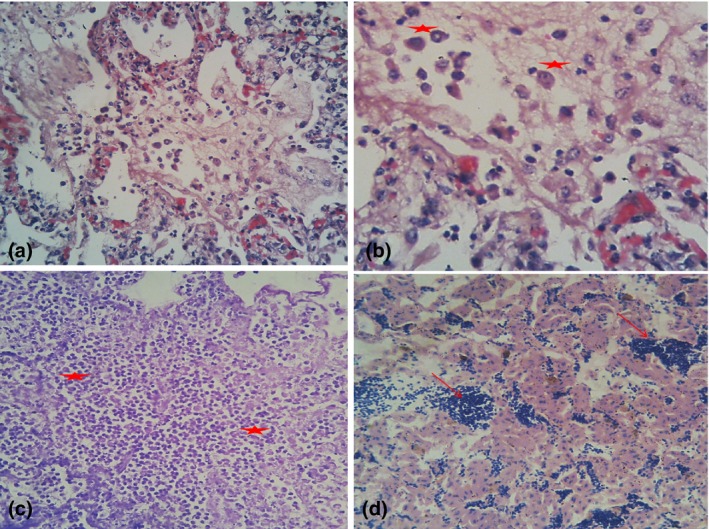
(a) Lung, Chicken; severe pulmonary oedema, parabronchial pneumonia and diffuse intraparabronchial infiltration of histio‐lymphoplasmacytic cells and fibrin in airsac. H&E X400. (b) Lung, Chicken; parabronchial and airsac infiltration by macrophages (heart failure cells), laden with intracytoplasmic hemosiderin. H&E X800. (c) Lung, Chicken; generalized severe parabronchial and airsac presence of numerous airsac macrophages (heart failure cells) admixed with lymphocytic cells. H&E X400. (d) Liver, Chicken; generalized severe coagulation necrosis, vascular congestion with sinusoids congested with autolytic erythrocytes (arrow). H&E X400.

Grossly, the crop and gizzard were filled with undigested guinea corn and rice grains. There was a garlic‐like odour of the crop contents. The trachea mucosa had a mucoid exudate that almost completely occluded it. Cyanotic combs and wattles in all the carcasses were seen. There was excessive abdominal fat and egg yolk peritonitis, and slightly pale carcass. Widespread congestion and petechial haemorrhages on the intestinal mucosa and caecal tonsils were seen, with presence of greenish‐yellow faecal material in the intestinal lumen. Congested heart, lungs, swollen and congested liver, enlarged and friable kidneys and enlarged spleens were also observed. A helminth worm was seen in one carcass. Microscopically, there was generalized severe pulmonary oedema, parabronchial pneumonia (Fig 1a) and diffuse intraparabronchial presence of air sac macrophages (Fig 1b,c). Also, the pulmonary parabronchial airway was infiltrated by lymphoplasmacytic cells and fibrin (Fig 1a). The hepatic cytoarchitecture showed generalized severe coagulation necrosis, vascular congestion and severe lymphocytic cellular infiltration (Fig 1d).

Based on the non‐specific lesions, differential diagnoses of poisoning, avian influenza, Newcastle disease and fowl typhoid were considered. Helminthosis due to the presence of helminth worms in the intestine was also considered.

### Toxicological analyses

Following a suspicion of poisoning, a toxicological approach to investigate possible zinc phosphide exposure was initiated. The silver nitrate test for phosphine gas was carried out as previously described (Mital *et al*. 1992) with slight modification. The crop content was collected, air‐dried and grounded. About 1 g of the pulverized sample was mixed with 5 mL of water in a test‐tube and 2–3 drops of concentrated hydrochloric acid was added. A 0.1 N silver nitrate impregnated paper strip was placed inside the test tube without touching the reaction mixture and then covered properly. The mixture was heated at 50°C for 15 min. The test is considered positive when the phosphine gas, liberated upon hydrolysis, turns the white silver nitrate paper strip to black. Further confirmation of phosphine was carried out by using lead acetate‐impregnated white paper strip which did not change colour thereby ruling out the presence of hydrogen sulphide gas that can also turn both paper strips to black. More confirmatory tests for phosphine gas was done by adding a drop of ammonium molybdate solution on the silver‐impregnated black paper strip, which turns to blue (Curry 1976). The suspected boiled rice sample was also subjected to the same tests as described above, and was returned positive. In addition to the above laboratory tests, samples submitted to bacteriology and virology laboratories returned negative results, while parasitology results revealed helminthic eggs in two samples.

### Confirmatory diagnosis

Based on the results obtained above, a diagnosis of phosphine poisoning was made.

## Discussion

Rodenticides are used for the control of rodent pests. Aluminium phosphide (AlP) and zinc phosphide are components of rat poison used as common outdoor and indoor pesticides or rodenticides in developing countries (Chugh 1992). Since the first available report of AlP poisoning in the early 1980s from India, it is now one of the most common causes of poisoning from agricultural pesticides (Gargi *et al*. 2006; Goel & Aggarwal 2007). Exposure of individuals occurs when the poisonous substance is inappropriately applied or wrongly used. In most cases, poisoning in humans and domestic animals is through accidental ingestion or deliberate use, which most of the times, requires forensic or medico‐legal analysis (Raina *et al*. 2003).

Exposure of chickens in the current report could possibly be linked to the outbreak of Lassa fever in humans in Nigeria with concomitant increase in sales of rodenticides, which possibly were used indiscriminately for the control of rodent vectors around homes or residential neighbourhoods (AFP 2016). The presence of widespread congestion and petechial haemorrhages on the intestinal mucosa, congested heart, lungs, swollen and congested liver, enlarged and friable kidneys and enlarged spleens with the microscopic pulmonary oedema and diffuse intraparabronchial presence of air sac macrophages (heart failure cells) strongly suggest an acute toxic cause of death, and is in line with earlier observations (Tiwary *et al*. 2005; Hakimoglu *et al*. 2015). Heart failure cells are haemosiderin‐containing macrophages generated in the alveoli of patients with chronic pulmonary oedema, when the high pulmonary blood pressure causes red cells to pass through the vascular wall. Also, the mucoid exudates on the trachea corroborated the pulmonary lesion. Chickens, especially those reared on free range system; feed on poisonous food materials meant for the control of rats and mice around the houses or neighbourhoods. The chemical substance in the rodenticide is activated and become poisonous when it reacts with hydrochloric acid in the stomach during the process of food digestion (Guale *et al*. 1994).

The sensitivity of silver nitrate test is very high even with low concentration of phosphine gas. Its specificity is also high except that sometimes, silver nitrate produces blackening due to reaction with hydrogen sulphide, but this was ruled out by reaction with lead acetate paper. However, gas chromatography with a nitrogen–phosphorous detector is the most sensitive and specific test; and this can be used for analysis of airtight samples (viscera and gastric contents) collected during autopsy (Tiwary *et al*. 2005). In the present case, the garlicky odour of crop contents, lesions observed at necropsy and histopathology and tests for phosphine gas in crop contents and rice samples, are confirmatory of phosphine poisoning in the chickens. Chickens have been reported to be acutely poisoned by zinc phosphide resulting in mortality (Tiwary *et al*. 2005). Other birds such as wild turkeys and geese are susceptible to zinc phosphide poisoning and have been reported to be intoxicated with the rodenticide (Poppenga *et al*. 2005; Bildfell *et al*. 2013).

The permissible exposure limit of phosphine is <0.3 ppm in the working environment and levels greater than 50 ppm are dangerous to life, while at 400–600 ppm it is lethal within half an hour (Sudakin 2005). Individuals working in the manufacturing facility of AlP or methamphetamine (phosphine is a by‐product), are at risk for unintentional exposure to phosphine gas, with few reported fatalities (Willers‐Russo 1999; Shadnia *et al*. 2008). Veterinarians, veterinary nurses and animal owners, who handle animals with phosphine poisoning can also be affected and sickened by phosphine gas (Easterwood *et al*. 2010).

The diagnostic approach described here is recommended for veterinary diagnostic laboratories where sophisticated chromatographic equipment is not available, and requires quick diagnosis where exposure to rodenticide is suspected in chickens and other domestic animals. Village chickens should be properly housed and provided with adequate shelter, feed, water and security. Baits or rodenticides and other obnoxious substances should be kept out of the reach of domestic animals, especially chickens. The rodenticides should be used appropriately for the control of pests.

## Source of funding

The current clinical case is not funded by any project grant.

## Conflict of interest

The authors declare that they have no financial or personal relationship (s), which may have inappropriately influenced them in writing this paper.

## Ethical statement

The authors confirm that the ethical policies of the journal, as noted on the journal's author guidelines page, have been adhered to and no ethical approval was required for this particular case report.
